# The Prognostic Value of MicroRNAs in Thyroid Cancers—A Systematic Review and Meta-Analysis

**DOI:** 10.3390/cancers12092608

**Published:** 2020-09-12

**Authors:** Cristina Alina Silaghi, Vera Lozovanu, Horatiu Silaghi, Raluca Diana Georgescu, Cristina Pop, Anca Dobrean, Carmen Emanuela Georgescu

**Affiliations:** 1 Department of Endocrinology, “Iuliu Hatieganu” University of Medicine and Pharmacy Cluj-Napoca, Victor Babes Street 8, 400012 Cluj-Napoca, Romania; Alina.Silaghi@umfcluj.ro (C.A.S.); lozovanu.vera9@gmail.com (V.L.); cgeorgescu@umfcluj.ro (C.E.G.); 2 Department of Surgery V, “Iuliu Hatieganu” University of Medicine and Pharmacy Cluj-Napoca, Victor Babes Street 8, 400012 Cluj-Napoca, Romania; horațiu.silaghi@umfcluj.ro; 3 International Institute for The Advanced Studies of Psychotherapy and Applied Mental Health, Babeș-Bolyai University, Republicii Street 37, 400015 Cluj-Napoca, Romania; 4 Department of Pharmacology, Physiology, and Pathophysiology, Faculty of Pharmacy, “Iuliu Hatieganu” University of Medicine and Pharmacy Cluj-Napoca, Louis Pasteur Street 6A, 400349 Cluj-Napoca, Romania; 5 Department of Clinical Psychology and Psychotherapy, Babeş-Bolyai University, Republicii Street 37, 400015 Cluj-Napoca, Romania; anca.dobrean@ubbcluj.ro

**Keywords:** thyroid cancer, papillary thyroid cancer, medullary thyroid cancer, microRNA, miRNAs, mir-146b, mir-221/222 cluster, biomarker, prognosis, survival, recurrence

## Abstract

**Simple Summary:**

Current prognostication systems have inherent limitations associated with the prediction of recurrence risk from thyroid cancer (TC). Recent studies identified associations between specific levels of microRNAs and aggressive TC clinicopathological features. The objective of this research was to uncover existing knowledge regarding dysregulated microRNAs in conjunction with the long-term prognosis in TC patients. We also set out to identify specific microRNAs that could serve as prognostic biomarkers in different subtypes of TC. The findings emerging from the meta-analysis revealed that elevated expression levels of miR-146b, miR-221, and miR-222 were significantly associated with recurrence and suggested their possible prognostic ability, especially in the subgroup of papillary thyroid cancer patients. All these results could aid decision-making for clinicians and optimize the surgical management of patients with TC. Also, they could help to refine the complex prognostication system and implement microRNA-targeted therapy of TC in the future.

**Abstract:**

Thyroid cancer (TC) includes various phenotypes, from indolent to highly aggressive cancer. The limitations of the current prognostication systems to predict the recurrence risk and the variability in expression of the genes involved in the thyroid carcinogenesis uncover the need for new prognostic biomarkers by taking into account potential epigenetic differences. We aimed to summarize the current knowledge regarding the prognostic impact of microRNAs (miRNAs) in TC. A literature search was conducted in PubMed, Embase, Scopus, and Web of Science databases. Both upregulated and downregulated miRNAs are significantly correlated with worse overall survival (hazard ratio (HR) = 5.94, 95% CI: 2.73–12.90, *p* < 0.001; HR = 0.51, 95% CI: 0.26–0.96, *p* = 0.048) disease/recurrence-free survival (HR = 1.58, 95% CI: 1.08–2.32, *p* = 0.003; HR = 0.37, 95%, CI: 0.24–0.60, *p* < 0.001). Sensitivity analysis revealed a significant association between the higher expression of miR-146b, miR-221, and miR-222 and the recurrence of papillary TC (OR = 9.11, 95% CI 3.00 to 27.52; *p* < 0.001; OR = 3.88, 95% CI 1.34 to 11.19, *p* < 0.001; OR = 6.56, 95% CI 2.75 to 15.64, *p* < 0.001). This research identified that miR-146b, miR-221, and miR-222 could serve as potential prognostic biomarkers in TC, particularly in PTC. Further studies are needed to strengthen these findings and sustain its clinical applicability.

## 1. Introduction 

Thyroid cancer (TC) represents 90% of endocrine tumors with an incidence that has been continuously rising and has more than tripled over the last four decades in the United States and many other countries [1]. It is the fifth leading cause of cancer in women, in line with the increase in global TC incidence [2]. Over 95% of TCs are derived from follicular epithelial cells and have been traditionally classified as well-differentiated thyroid carcinoma (DTC). The DTC comprises two main histological entities, the follicular (FTC) and the papillary (PTC) thyroid carcinomas, which following dedifferentiation are assumed to generate the poorly DTC (PDTC) and the highly aggressive anaplastic thyroid carcinomas (ATC) [3,4]. Medullary thyroid carcinoma (MTC) is a neuroendocrine malignancy of parafollicular C cells that occurs in sporadic (75%) and hereditary (25%) types [5]. The overall outcome of TC is generally favorable. However, a minority of patients follow a more aggressive clinical course and may lead to tumor recurrence or death in the subsequent five years [6]. 

Several clinicopathological factors have proven useful in distinguishing patients who have a low risk of recurrence or death from those with intermediate to high risk. These include stage and age at diagnosis, tumor size and differentiation status [7], the presence of extrathyroidal extension [8], lymph node metastases, vascular invasion [9], radioiodine resistance [10], and specific histopathological variants [11]. Many of the factors are strongly inter-related and are not independent predictors of patients’ outcomes. Accordingly, it led to the development of several scoring systems, of which the American Joint Committee on Cancer/Union for International Cancer Control Tumor-Nodule-Metastases (AJCC/UICC TNM) staging system outperforms other systems in terms of survival prognosis [12,13]. However, the AJCC/UICC TNM system has inherent limitations associated with the prediction of recurrence risk from TC [14]. This inaccuracy may be related to the failure of current staging systems to adequately integrate the risk associated with other potentially important features, such as molecular profile.

In the last few years, various molecular markers have been investigated as prognostic biomarkers of poor outcomes for TCs. In particular, activating mutations of BRAFV600E [15], RAS, and TERT promoter genes [16], and rearrangements of RET/PTC [17] and PAX8/PPARγ genes [18] are known to be associated explicitly with TC initiation and progression [19]. However, not all aggressive tumors exhibit these genetic variations [20], and several genes exhibit an altered expression that cannot be attributed to mutations, rearrangements, or amplification, but they rather are epigenetically dysregulated [21], as evidenced recently by bioinformatic analysis that evaluated the global gene expression pattern in thyroid cancer subtypes and identified their differentially expressed genes [22]. They can serve as markers of prognostic significance.

The epigenetic profile of TC, namely alterations in microRNAs (miRNAs) expression, has been determined to modulate gene expression [23]. MiRNAs are a class of non-coding RNAs approximately 19–24 nucleotides in length that can function as oncogenes or tumor suppressors by inhibiting the translation of tumor suppressor genes or blocking the translation of oncogenes, respectively [23]. Such activities have been demonstrated under normal human physiological conditions and implicated as contributors to the pathological process of carcinogenesis [24]. 

Genome-wide analyses have generated specific miRNA profiles of different histotypes of TCs and identified the upregulated and downregulated miRNAs related to various carcinogenesis stages and prognoses. Most miRNAs studies concern PTC, which is the most common type of DTC with excellent 5-year survival. However, in up to 5–10% of cases, PTC patients experience a more aggressive clinical course, which is characterized by early metastases, increased mortality, resistance to radioactive iodine, and disease recurrence [25,26]. A characteristic miRNA signature associated with PTC involves the overexpression of miR-146b, miR-221, miR222, miR-21, and miR-181b [27,28,29], and downregulation of let-7f [30].

In particular, miRNA 146b is predominantly overexpressed in PTC [27,31]. It is associated with a more aggressive phenotype, BRAF mutations, extrathyroid invasion, advanced stages of the disease, and a poorer prognosis [32,33]. Similar results have been found in other studies for the miR-221 and miR-222 families, which are overexpressed in tumors with worse prognostic characteristics such as increased tumor size, capsular, vascular or lymphatic invasion, or the presence of metastases [34,35]. 

The plasma levels of miR-222 and miR-146b were higher in PTC patients when compared with the plasma of healthy volunteers, and their levels dropped to similar levels of healthy subjects after total thyroidectomy [25]. These findings raised the potential of using miRNAs as a noninvasive, alternative recurrence surveillance tool. However, the majority of studies have measured miRNAs expression in thyroid tumor tissue and remains unclear whether circulating miRNAs levels can accurately reflect miRNAs expression in specific tissues [36]. 

Studies on FTC patients found a limited set of deregulated miRNAs, specifically the overexpression of miR-197 and miR-346 [37].

ATC as the most aggressive subtype of TC is the least responsive to therapy and has a poor clinical outcome due to fast-growing tumors and metastatic spread [23,38]. Analysis of miRNAs expression in ATC demonstrated a decreased expression of miR-30, miR-26, miR-125, miR-92, and let-7, together with an increase in miR-21, miR-146, miR-221, miR-22, miR-17, and miR-19 levels [27,31,39,40].

MTC accounts for 3% of TC incidence. Its prognosis is not as favorable as DTC, with a reported 10-year survival rate of 65% overall [41]. The overexpression of miRs-183 and 375 in MTC are predictors of lateral lymph node metastases, residual disease, distant metastases, and mortality [42].

A previous systematic review of the literature was conducted to examine the associations between expression levels of specific miRNAs and aggressive clinicopathologic features in PTC [43]. However, to our knowledge, there is no published systematic review examining the miRNAs expression role as prognostic biomarkers in the long-term surveillance of TCs. The objective of this systematic review and meta-analysis is to summarize the current knowledge regarding dysregulated miRNAs and to evaluate their prognostic impact in patients with TC.

## 2. Methods

### 2.1. Protocol and Registration

The protocol of the current systematic review is available and can be accessed on the Prospero website https://www.crd.york.ac.uk/prospero/ with the following registration number: 173854.

### 2.2. Search Strategy

The research study followed the Preferred Reporting Items for Systematic Reviews and Meta-Analyses (PRISMA) guidelines [44]. We used the PICOT (population, index, comparator, outcome(s), timing, setting) system to describe the essential items for framing this review and its objective and methodology. Papers published before 10 January 2020, were searched on the PUBMED, EMBASE, Web of Science, and Scopus databases, and other sources (EthOS, Explore at the British Library, OpenGrey, Grey Literature Report, Clinicaltrials.gov, Google Scholar). We combined the concepts “thyroid carcinoma” with “microRNA” by using “AND” as the Boolean operator. After that, we developed the following search strategy on Medline: ((“thyroid neoplasms” [MeSH] OR “thyroid cancer*” OR “thyroid carcinoma*” OR “thyroid malign*” OR “thyroid neoplas*”) AND (microRNA* OR miRNA* OR miR*)). The search strategy in other databases was similar, following the same principles. Simultaneously, the reference lists of review papers and original reports were hand-searched for further relevant studies. 

#### 2.2.1. Inclusion Criteria

To be included in the analyses, studies had to meet the following criteria:

1. Longitudinal studies aiming to investigate the prognostic value of microRNA expression in TC patients;
2. Studies with the following types of outcome available: overall survival (OS), tumor-specific survival (TSS), disease-free survival (DFS), recurrence-free survival (RFS), distant metastases-free survival (DMFS), progression-free survival (PFS); parameters of complicated course of the disease, such as residual, persistent, recurrent, and progressive disease;
3. Studies with a minimum follow-up period of 12 months for the outcome of interest;
4. Studies offering enough information to compute effect size;
5. The full-text paper was available in English, French, or Russian (languages known by the authors).

#### 2.2.2. Exclusion Criteria Included the Following:

1. Studies about microRNAs expression in patients with other types of malignancies;
2. Studies of participants with TC from diseases predisposing to malignancy;
3. Review articles (narrative reviews, systematic reviews, and meta-analyses), letter to editor and correspondence without original data, dissertations and conferences abstracts;
4. Full texts unavailable for review.

Two researchers screened all abstracts and flagged potentially eligible studies (L.V., S.C.A.). Then, these were retrieved full-text and independently assessed against the inclusion/exclusion criteria by the same researchers. Any disagreements were resolved by discussion with a third author (S.H.).

### 2.3. Data Extraction

The outcomes of interest were OS, TSS, DFS, RFS, DMFS, PFS, and parameters of complicated course of the disease, such as residual, persistent, recurrent, and progressive disease. For each study included, two independent researchers (L.V., S.C.A.) extracted the following information:

1. Publication information (first author, year of publication, country of origin),
2. Patients’ characteristics (number of participants, age, histopathological typing of TC);
3. miRNA detection information (miRNA type, sample type, expression status, assay type, cut-off values, normalization control);
4. Prognosis information (the reported outcome, follow-up timing);
5. Data for computing the effect size (hazard ratio (HR) or odds ratio (OR) with corresponding 95% confidence interval (CI) and log-rank P-value, reported directly or means, standard deviations, and sample size).

In those cases where the research question of the primary studies was broader than the one of the current analyses, we extracted only data necessary for computing the effect size (ES). Where there was insufficient information to compute the ES, we contacted the corresponding authors of the original studies. 

### 2.4. Assessment of Methodological Quality

The Quality Assessment of Prognostic Accuracy Studies (QUAPAS), an extension of the Quality Assessment of Diagnostic Accuracy Studies (QUADAS-2) tool, supplemented by elements from the Quality in Prognosis Studies tool (QUIPS) [45,46,47] was used by two independent researchers (L.V., S.C.A.) to assess the quality of the included studies. QUAPAS assesses the methodological quality of the studies in two areas: (a) risk of bias and (b) applicability concerns. 

The researchers evaluated the risk of bias in five domains: (1) patients’ selection, (2) index test, (3) target event, (4) study flow, and (5) analysis. Each domain contains two to four signaling questions, which were adjusted to the current review subject (see Appendix A, Table A1). They are answered as “yes”, “no”, or “unclear”, and they are phrased such that “yes” indicates a low risk of bias. The risk of bias is judged as “low”, “high”, or “unclear”. If all signaling questions for a domain are answered “yes”, then the risk of bias will be judged as “low”. If any signaling question is answered “no”, this flags a high risk of bias. The “unclear” category was used only when insufficient data are reported to permit a judgment. 

The applicability section is structured in a similar way to the bias section, but it does not include signaling questions and involves only the first four domains. Concerns regarding applicability are rated as “low”, “high”, or “unclear”. Again, the “unclear” category was used only when insufficient data were reported.

Finally, to evaluate the robustness of the analyses, we performed a sensitivity analysis, including only studies rated with a low risk of bias in at least three of the five domains.

### 2.5. Statistical Methods

The Comprehensive Meta-Analysis (CMA v. 2.2.064) and Stata SE 16.1 (STATA Corp., Inc., College Station, TX, USA) software, Admetan package was used to compute the ESs and to generate the forest plots. Before calculating the ESs for each outcome, we performed the log transformation of the row data, as the log transformation makes the scale and the associated 95% CIs symmetric [48]. We calculated the ESs as HR and OR for each outcome expressing the association of miRNAs with OS, DFS/RFS, distant and regional recurrence, respectively, residual/persistent disease. When possible, the HR and OR and the associated variance were obtained directly from the primary studies; otherwise, we based our estimations on alternative statistics such as means, standard deviation, and the total sample size [49]. We also conducted sensitivity analyses by excluding those studies ranked as a high risk of bias and for studies assessing only specific miRNAs such as mir 146b, mir 221, or mir 222. A value greater than 1 for the pooled ESs suggested poor prognosis, while a value below 1 suggested a favorable prognosis. 

Moreover, as we observed high heterogeneity between studies due to large numbers of miRNAs evaluated in primary studies, the random-effect model (the DerSimonian and Laird method) was used [50]. We assessed the percentages of heterogeneity by using the I^2^ index. I^2^ indicates the percentage of variance in effect sizes attributable to true, between-study heterogeneity rather than sampling error or chance. I^2^ values of around 25%, 50%, and 75% were considered indicators of low, moderate, and high heterogeneity, respectively [51]. In addition, we calculated 95% CIs around I^2^ using the non-central χ^2^-based approach with the heterogi module for STATA [52]. We set the statistical significance threshold for all analyses at 0.05. 

### 2.6. Ethical Approval

This article does not contain examinations performed on human participants. Then, ethical approval was not necessary.

## 3. Results

### 3.1. Literature Search

**Figure 1:**
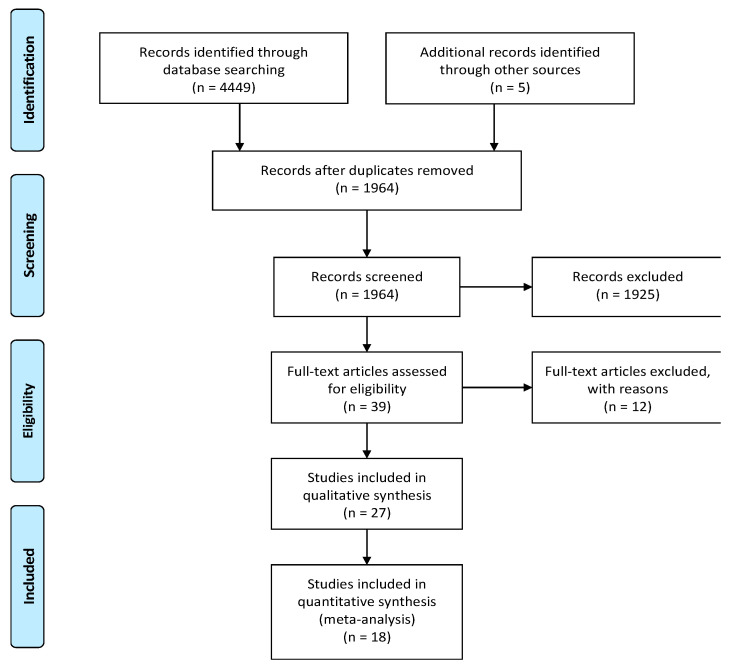
Preferred Reporting Items for Systematic Reviews and Meta-Analyses (PRISMA) flow-chart.

Our literature search in PUBMED, EMBASE, Web of Science, and Scopus databases until January 10, 2020 identified 4459 potentially relevant publications. We included an additional five studies from other sources (EthOS, Explore at the British Library, OpenGrey, Grey Literature Report, Clinicaltrials.gov, Google Scholar). After removing duplicates, 1964 abstracts were identified. A total of 1925 records were excluded as they represented irrelevant studies to the current analysis, review articles, letters or reply, in vitro studies, diagnostic studies, and bioinformatics studies. The remaining 39 articles were deemed relevant by title and abstract alone. Based on the readings of the full-text articles, we excluded 12 articles for the following reasons: no or insufficient follow-up time (*n* = 2), studies evaluating microRNA polymorphisms (*n* = 2), did not explain the prognostic value of microRNA (*n* = 2), no full-text available (*n* = 4), patients originated from The Cancer Genome Atlas (TCGA) database (*n* = 1), unavailable statistical analysis (*n* = 1). Finally, 27 articles met the initial eligibility criteria and were systematically reviewed and abstracted, of which 18 studies were included in the quantitative analysis. Figure 1 presents a flow diagram of the study selection process. 

### 3.2. Participant and Study Characteristics

A total of 27 articles from 10 countries with 78 assessments of association between cancer prognosis and 39 different miRNAs were included in the review [42,53,54,55,56,57,58,59,60,61,62,63,64,65,66,67,68,69,70,71,72,73,74,75,76,77,78]. Table 1 summarizes the characteristics of the included studies. The authors published all 27 articles in English. The year of publication ranged from 2011 to 2019. The total number of patients included was 2011, ranging from 8 to 165 patients per study. The average age of participants of the 20 articles that reported the mean or median values is 51.1 years and ranges between 42 and 71.5 years among individual studies. The authors provided information on the gender of the included participants in 25 studies; the average female percentage is 63.7%. The included studies were conducted in a variety of countries, including China, Italy, Israel, Australia, Spain, Brazil, the United States, Japan, Switzerland, and Taiwan. The dominant ethnicity was Asian in more than half of the studies, while the second most dominant was Caucasian. Most studies were retrospective in design. 

Concerning histological subtypes, the most common evaluated was PTC, which was found in 19 studies, followed by medullary thyroid cancer (MTC), assessed in 6 studies [42,53,60,67,68,78] and minimally invasive follicular thyroid cancer (MI-FTC) found in one study [63], respectively. One study included all subtypes of well-differentiated TC (DTC), and one study evaluated patients with poorly differentiated TC (PDTC) [55,56]. From all MTC patients, the average percentage of sporadic MTC ranged from 57% to 89% among individual studies. The patients in the included studies were clinically staged I–IV according to the AJCC/TNM staging system in 26 studies, while one study recruited participants with stage II and III [56]. The authors specified the initial curative treatment in 23 studies, being represented by total or subtotal thyroidectomy in all cases. Some patients underwent an additional lymph node dissection or adjuvant radioactive iodine (RAI) ablation therapy in 4 and 9 studies, respectively. 

For initial biomarker screening, six studies performed miRNA microarray [42,54,56,64,76,78], one study performed quantitative PCR-based array [63], and one study performed MiRNome sequencing [55], respectively, followed by qualitative reverse transcriptase PCR (qRT-PCR) to validate the results. Conversely, 20 studies identified candidate miRNAs from the literature and performed qRT-PCR after that. The other two studies performed either MiRNome profiling or microarray without subsequent PCR validation [55,56]. The expression of miRNAs was mainly measured in tumor tissues, while five studies examined miRNAs in serum or plasma [62,73,74,75,78]. The study authors described the endogenous control for normalization of the RNA input in 26 studies. The most commonly used endogenous control was RNU6, which was found in 10 studies [54,57,58,59,66,67,70,71,72,75]. The second most used were RNU48 and RNU6B, each being found in four studies [42,53,60,61,64,68,77,78]. RNU44 and GAPDH were found in two studies each [63,65,74,76]. 

Dettmer at al. used both RNU44 and RNU6 [56]. The other two studies used either mir-16 or b-actin as endogenous control [69,73]. Notably, of the studies that set the cut-off values of miRNAs expression, seven studies reported median values [55,58,61,62,72,77,78], and three studies used means [70,71,75]. Two studies set the miRNA threshold by using the receiver operating characteristic (ROC) curve [66,74], and miRNA fold-change was used in one study [56], respectively. The other 14 studies did not set a cut-off; instead, they calculated the mean miRNA expression between the participants with a different outcome. 

In terms of participants’ surveillance, the average follow-up duration of the 20 studies that reported the mean or median values is 62.1 months, ranging from 12 to 127 months in the pooled studies. Eight studies did not provide the mean follow-up directly. Therefore, it was extracted from the Kaplan–Meier curve with the range from 60 to 202 months.

Concerning outcomes, which are described in detail in the sections below, 16 records focused on survival analysis: overall survival (OS) [57,59,62,65,69,70,71,72,73,75,78], tumor-specific survival (TSS) [56], disease-free survival (DFS) [55,56,58,75], or recurrence-free survival (RFS) [61,74,77] and another 14 records evaluated different parameters of long-term poor prognosis, i.e., regional and/or distant recurrence [42,54,63,64,66,69,73,76], persistence [53,67,68], progression [53,59,60], and residual disease [42,55,78].

Concerning the statistical analysis of the included articles, there was a high variability in the analytical data obtained from the different records. The HR and corresponding 95% CI set as the primary index of the effect size was reported in 11 studies [56,58,59,61,62,65,72,74,75,77,78] and estimated from the χ^2^ and log-rank p-value in one study [55]. 

Among those mentioned above, five studies performed multivariate analysis and reported adjusted HR results of the outcomes [56,58,65,72,75]. Several studies did not report the HRs and 95% CIs, and we reported their results narratively [57,69,70,71]. ORs and corresponding 95% CIs, set as the other ES, were reported directly in one study [53]. Otherwise, we estimated the ES from means and standard deviations (medians and interquartile ranges) of miRNA expression levels in the compared groups or p-values and sample sizes in 14 studies.

Three studies did not report data for the ES calculation but were still included in this review as they fit our inclusion criteria [21,28,35]. However, it has not been possible to obtain the relevant information; therefore, they are reported narratively in this review. We contacted 8 authors by email; none of them replied and provided any relevant information [53,57,66,69,70,71,79,80]. We could not perform several planned subgroup and sensitivity analyses, based on study design, population demographics, AJCC/TNM disease stage, types of biological samples, mutational status, and initial curative therapy, due to the limited reporting of studies and a small number of studies.

### 3.3. Excluded Studies

A total of 12 studies were excluded, with reasons [79,80,81,82,83,84,85,86,87,88,89,90]. Specifically, two studies evaluated miRNA polymorphisms [89,90], and two studies reported data other than the prognostic role of miRNAs [79,87,88], which was not the topic of this review. One study reported a duration of follow-up of less than 12 months [86]. The authors of one study extracted the patients’ data from the TCGA database; for this reason, it was excluded [80]. For five studies, only a conference abstract was available, and no full-text article was published [81,82,83,84,85]; therefore, they were excluded.

### 3.4. Quality Assessment

**Figure 2:**
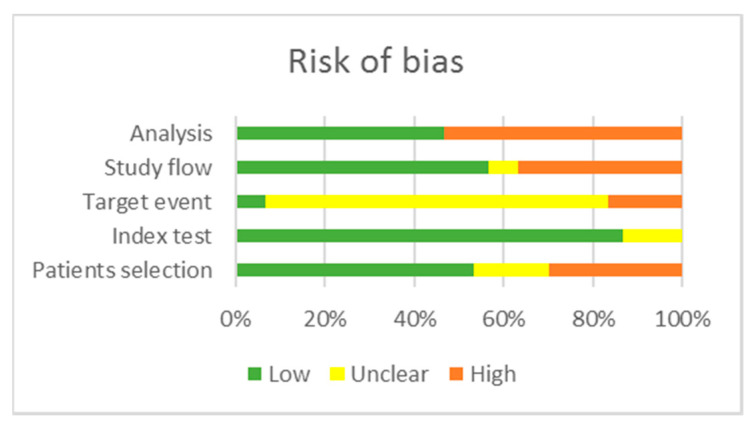
Risk of bias of the included studies.

**Figure 3:**
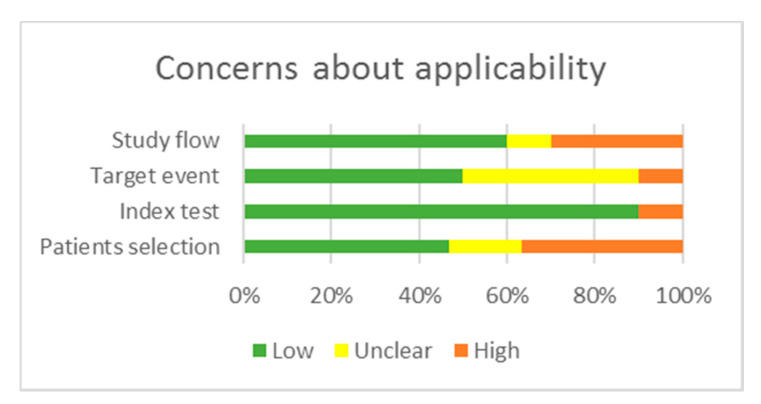
Concerns regarding applicability.

Two reviewers (S.C.A. and L.V.) critically assessed the quality of the 27 studies included in the qualitative analysis using the QUAPAS tool. We used graphs (Figure 2 and Figure 3) and a table (Appendix B, Table A2) to present results for the risk of bias and applicability concerns of each domain). Since many studies included multiple target events and statistical analysis, we divided them into two groups: studies reporting time to event outcomes and studies reporting other parameters of long-term, complicated course of the disease; thus, three studies [55,69,78] are included in both categories, raising the total number of appraisals to 30. We determined a high risk of bias for the “Patient selection” domain in the pooled studies, due to inappropriate enrolment or exclusion in several articles [54,55,56,57,58,59,63,70,72,73,74,76,77,78]. Almost all records scored a low risk of bias for the “Index test” domain as the methods of conducting and interpreting the miRNA measurement had been valid, reliable, and identical for all participants. The overall risk of bias concerning the target event was labeled as unclear, because the definition, the method of outcome assessment, and whether the evaluators were blind to the index test results had been poorly described. The pooled risk of bias for study flow was set as low; albeit, there was noted missing information about the excluded or lost to follow-up patients in 13 articles [42,53,54,55,57,58,59,62,69,70,71,72,78], which therefore scored high or had an unclear risk of bias. In the domain “Analysis”, 15 articles scored high on the risk of bias as they did not account for censoring, competing events, or reported just hypothesis testing, without an estimation of effect magnitude [42,53,54,55,57,60,63,66,67,68,69,70,71,73,78].

Regarding applicability, there are some concerns about the “Patients selection” domain in 6 manuscripts as the cohorts comprised cases with a higher percentage of AJCC/TNM advanced stages compared to general statistics [65,66,70,71,72,74]. There is an unclear concern about the applicability in the “Target event” domain, as 12 articles described the outcomes insufficiently [54,55,57,59,62,69,70,71,72,74,75,76]. The overall methodological quality of the included studies was judged as moderate for studies reporting time-to-event outcomes and low for studies reporting other parameters of aggressive disease behavior.

To explore the influence of the studies’ quality on the results, we performed sensitivity analyses for studies rated with the lowest risk of bias, instead of excluding the studies with an unclear or high risk of bias.

### 3.5. Key Results Regarding miRNAs and Prognosis

The increased expression of 21 miRNAs (miR-10b, miR-1299, miR-146a, miR-146b, miR-150, miR-155, miR-15a, miR-182, miR-183, miR-193b, miR-19b, miR-200a, miR-23b, miR-203, miR-220, miR-221, miR-221*, miR-222, miR-222*, miR-375, miR-92a) and the decreased expression of 17 miRNAs (miR-1, miR let 7a, miR-9, miR-10b, miR-26a, miR-31, miR-34b, miR-130-b, miR-138, miR-139-5p, miR-199a-3p, miR-224, miR-381, miR-483-5p, miR-486, miR-791, miR-1271) was correlated with at least one endpoint of poor prognosis in TC (see Table 2 and Table 3). Inconsistent expression direction among studies was noted in regard to miR-21 [54,61,68,77] (see Table 4). The miRNAs evaluated in at least two studies were miR-146b, miR-155, miR-21, miR-26a, miR-203, miR-221, miR-222, miR-224, and miR-375. The most studied miRNA, miR-146b, was found in seven articles that described the association between tissue and serum miR-146b overexpression and poor prognosis in PTC, of which four studies compared miR-146b expression between recurrent and non-recurrent participants [64,69,73,76], two studies reported RFS [61,77], one article provided DFS [58], and OS was found in a paper [69], respectively. Secondly, the tissue and circulating expression profile data of the miR-221/222 family members, miR-221, and miR-222-predicted recurrence of the follicular cell-derived DTC (PTC, FTC) in 4 studies [63,64,73,76], respectively. Secondly, the tissue and circulating expression profile data of the miR-221/222 family members, miR-221, and miR-222 predicted recurrence of the follicular cell-derived DTC (PTC, FTC) in 4 studies [63,64,73,76].

#### 3.5.1. Dysregulated miRNAs Correlated with Survival Outcomes

A total of 16 records reported survival endpoints as time-to-event outcomes, involving 18 miRNAs. 

Overall survival (OS), defined as the time from initial treatment to death from any cause, was assessed in 11 studies. The high expression of four miRNAs (miR-146a, miR-146b, miR-182, miR-203) [69,72,74] and the low expression of six miRNAs (miR-1271, miR-791, miR-381, miR-let 7a, miR-26a, miR-486) [57,59,62,65,70,71,75,78] was correlated with decreased OS in PTC. Additionally, the high expression of miR-375 correlated with decreased OS in MTC [78].

Tumor-specific survival (TSS) is defined as the time from initial treatment to death from TC, and it was reported in one study, in which PDTC subjects with high levels of miR-150 had a significant decrease in TSS [56]. 

Disease-free survival (DFS) is defined as the time from initial therapy until disease recurrence or death from any cause, and it was evaluated in four studies. Therefore, the overexpression of miR-146b, miR-23b [56,58], and the down-expression of miR-139-5p and miR-26a, respectively, were correlated with decreased DFS [55,75].

Recurrence-free survival (RFS) is defined as the time from initial treatment to the first detected recurrence or the last follow-up visit without recurrence and it was evaluated in 3 studies, in which the higher expression of miR-146b, miR-203, miR-220, miR-221, and miR-222 [61,74] correlated with shorter RFS. Additionally, the low expression of miR-9, miR-10b, and miR-21 correlated with worse RFS of lymph node metastases [77]. 

However, most of the articles poorly defined the survival endpoints, and none reported PFS or DMFS.

#### 3.5.2. Dysregulated miRNAs Correlated with TC Recurrence

A total of eight studies reported 14 miRNAs that were differentially expressed between samples of recurrent and non-recurrent patients [42,54,63,64,66,69,73,76]. Abraham et al. [42] defined recurrent disease as a clinical and biochemical cure at three months but disease recurrence after that. Dai et al. [61] defined recurrence according to clinical, laboratory, and radiological evidence. Two studies defined recurrence-free status as no evidence of disease on clinical, laboratory, and radiological exams after follow-up [74,77]. Huang et al. defined metastatic recurrence as any distant metastasis recognized after the initial operation [62]. However, this endpoint differs between studies, and it is poorly defined or absent in most of the articles. 

The recurrence of PTC, evaluated in five studies, was correlated with the upregulation of 11 miRNAs (miR-15a, miR-19b, miR-21, miR-146a, miR-146b, miR-155, miR-193b, miR-200a, miR-221, miR-222, and miR-1299) [54,64,69,73,76] and the downregulation of six miRNAs (miR-1, miR-31, miR-34b, miR-130b, miR-138, and miR-483-5p) [54,64,69,72,73,76], respectively.

The regional recurrence is a term offered for the recurrence of regional lymph nodes, and it was reported in two studies and was correlated with the up-regulation of miR-183 and miR-375 in MTC patients [42], and downregulation of miR-199a-3p in PTC [66], respectively.

The distant recurrence, evaluated in two studies, was correlated with the up-regulation of miR-183 and miR-375 in MTC patients [42], and seven miRNAs (miR-10b, miR-92a, miR-221, miR-221*, miR-222, miR-222*, miR-375) in minimally invasive FTC subjects [63].

#### 3.5.3. Dysregulated miRNAs Correlated with TC Progression, Persistence, and Residual Disease

The progressive disease status of MTC was appraised in two studies by measuring the doubling time Ctn and CEA, with a cut-off of 24 months and imaging techniques to measure the variation in the size of lesions following RECIST criteria. A high expression of miR-375 and low expression of miR-224 were correlated with the progressive disease status [53,60]. No studies were reporting the correlation between miRNAs dysregulation with other histological subtypes of TC.

The persistent disease status of MTC was evaluated in three studies by dividing the subjects in the persistent and biochemically cured group. Therefore, the patients would be considered biochemically cured if the Ctn level was less than 10 pg/ml one year after primary surgery or at the latest follow-up. A high expression of miR-21 and low expression of miR-224 correlated with persistent disease status [53,67,68]. However, none of the studies reported this outcome in conjunction with other histological subtypes of TC.

Dysregulated miRNAs, in conjunction with the MTC residual disease, were approached in three studies [42,55,78]. Residual disease was explicitly defined as the outcome of interest in a study, representing cases with either persistent disease (high calcitonin three months after initial surgery) and with recurrent disease (clinical and biochemical cure at three months but disease recurrence after that) [42]. Therefore, the other two studies analyzed patients with persistent or recurrent disease, which can be assigned to the residual disease outcome, are included in the analysis [55,78]. The residual disease status was correlated with a high expression of miR-375 and miR-183 in MTC and low expression of miR-139-5p in DTC [42,55,78].

### 3.6. Meta-Analysis

#### 3.6.1. The Meta-Analysis by the Type of miRNAs Deregulation

##### Upregulated miRNAs

**Figure 4:**
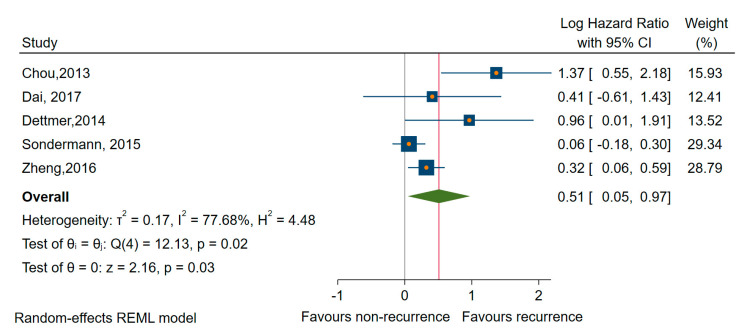
Forest plot of the association between the upregulates miRNAs and recurrence.

As shown in Table 2, a total of 14 studies investigated the association of up-regulated miRNAs and prognostic outcomes [42,56,58,61,63,64,68,69,72,73,74,76,77,78]. Due to the large percentages of heterogeneity between studies observed, we employed the random-effects model for each analysis. Thus, regarding recurrence, six studies looked specifically at the association of the up-regulated expression and rates of distant and/or regional recurrence, with a pooled OR of 8.44 (95% CI 3.24 to 21.98; log OR of 2.13, 95% CI: 1.20 to 3.07, *p* < 0.001), and a high percentage of heterogeneity (I^2^ = 64%, 95% CI: 18 to 86) (Figure 4). The miRNAs involved were miR-10b, -92a, -183, -146a, -146b, -155, -183, -193b, -221, -221*, -222, -222*, -375, and -1299). To evaluate the robustness of our analyses, we had performed the estimations only for studies with a low risk of bias in at least three domains [64,76], and we found no changes regarding the recurrence rate (all miRNAs, OR of 2.97, 95% CI 1.11 to 7.93; log OR = 2.10, 95% CI 0.040 to 4.150, *p* = 0.0046). 

**Figure 5:**
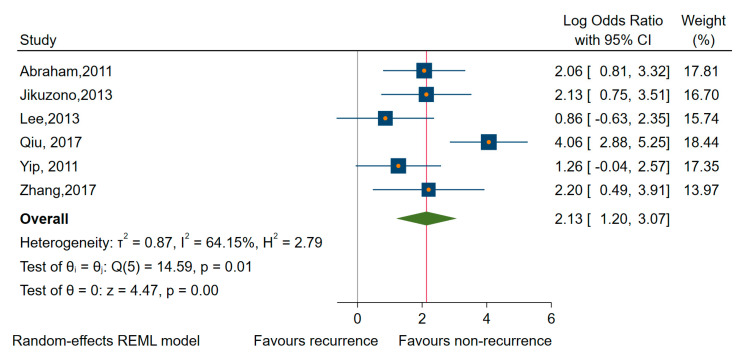
Forest plot of the association between the upregulated miRNAs and DFS/RFS.

Considering studies reporting DFS/RFS [56,58,61,74,77], we found that higher expressions of miRNAs (miR-23b, -146b, -150, -203, -220, -221, and -222) were significantly associated with worse prognosis (HR of 1.58, 95% CI 1.08 to 2.32; log HR = 0.51, 95% CI 0.05 to 0.97, *p* = 0.003) and large amounts of heterogeneity, I^2^ = 78% (95% CI 14 to 87) (Figure 5). We found similar results when we estimated only for studies rated with a low risk of bias in at least three domains [56,58,61,77], HR of 1.90, and 95% CI 0.93 to 3.82 (log HR = 0.64, 95% CI: 0.06 to 1.34), although the association became non-significant, *p* = 0.074. 

**Figure 6:**
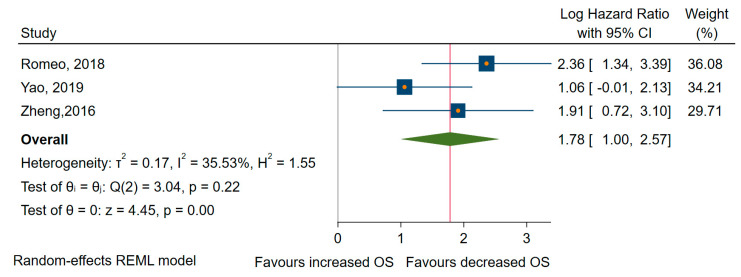
Forrest plot of the association between the upregulated miRNAs and OS.

Finally, concerning OS, three studies revealed that miRNAs overexpression was significantly associated with shorter OS [72,74,78], with an HR of 5.940, 95% CI 2.73 to 12.90 (log HR = 1,78, 95% CI: 1.00 to 2.57, *p* < 0.001), and a medium amount of heterogeneity, I^2^ = 34% (95% CI: 0 to 78) (Figure 6).

##### Downregulated miRNAs

**Figure 7:**
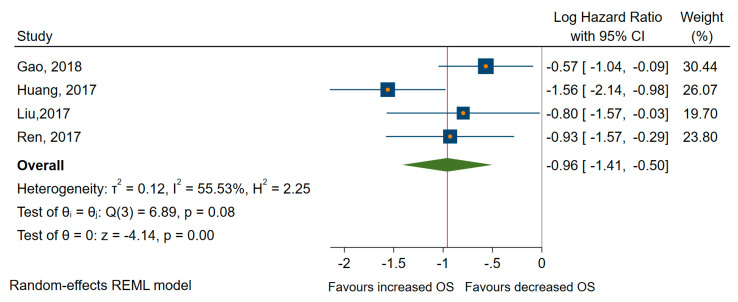
Forest plot of the association between downregulated microRNAs (miRNAs) and OS.

From all the studies included in meta-analyses, seven pieces of research looked at the low expression of miRNAs as predictors of long-term outcome in TC patients with high heterogeneity between studies [55,59,61,62,65,75,77]. Thus, the random-effects model was applied in subsequent analyses. From all studies, four papers examined the specific relationship with the OS [59,62,65,75]. All miRNA involved (miR-26a, -381, -791, -let 7a) were significantly associated with better OS, with a pooled HR of 0.37, 95% CI 0.24 to 0.60 (log HR = −0.96, 95% CI: −1.41 to −0.50, *p* < 0.001) and a medium amount of heterogeneity, I^2^ = 56% (95% CI 0 to 86) (see Figure 7). 

**Figure 8:**
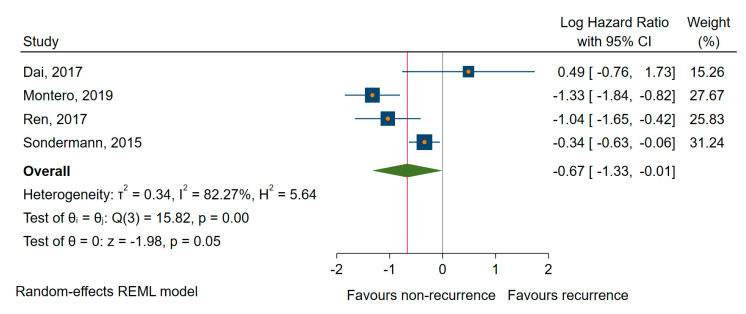
Forest plot of the association between the downregulated miRNAs and DFS/RFS.

We found similar results for DFS/RFS; four studies [55,61,75,77] showed a significant association between the downregulated miRNAs (miR-9, -10b, -21, -26a, and -139-5p) and shorter DFS/RFS, with a pooled HR of 0.51, a corresponding 95% CI 0.27 to 0.97 (log HR = −0.67, 95% CI: −1.33 to −0.001, *p* = 0.048), and a large amount of heterogeneity, I^2^ = 82% (95% CI 60 to 94) (Figure 8). In the sensitivity analysis, the exclusion of Montero et al. [55], for which we estimated the HR and 95% CIs from other provided calculations, the analysis (leave-one-out meta-analysis) relieved a non-significant association (*p* = 0.173). 

#### 3.6.2. Sensitivity Analysis by Specific Types of miRNA 

**Figure 9:**
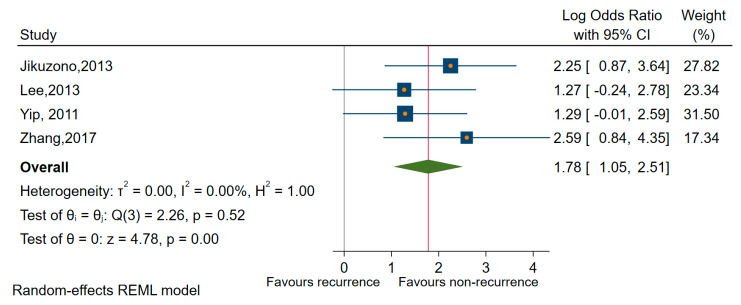
Forest plot of the association between miR-221/222 cluster expression and recurrence.

The amount of heterogeneity in the analysis of upregulated miRNAs and recurrence was dampened in sensitivity analyses when we estimated the pooled OR only for miR-221 (OR of 4.67, 95% CI 2.03 to 10.70; log OR = 1.54, 95% CI: 0.73 to 2.35, *p* < 0.001), I^2^ = 19% (95% CI 0 to 88) and for miR-222 (OR of 7.63, 95% CI: 3.64 to 15.96; log OR = 2.03, 95% CI: 1.29 to 2.77, *p* < 0.001), I^2^ = 0% (95% CI: 0 to 85) as well as for miRNAs of 221 and 222 as a family (OR = 5.924, 95% CI: 2.85 to 12.29; log OR = 1.78, 95% CI: 2.05 to 2.51, *p* < 0.001), I^2^ = 0% (95% CI: 0 to 85) in conjunction with recurrence in all types of TC reported (PTC, MI-FTC), [63,64,73,76] (Figure 9). 

**Figure 10:**
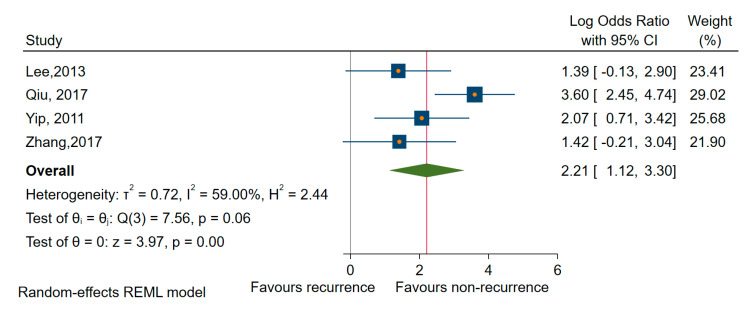
Forrest plot of the association between miR-146b expression and recurrence.

Estimations for mir-146b revealed a significant association with recurrence in PTC patients, which was the only subtype of TC reported in the four studies involved [64,69,73,76] (OR = 9.11, 95% CI: 3.00 to 27.52; log OR = 2.21, 95% CI: 1.12 to 3.30, *p* < 0.001), while the amount of heterogeneity, I^2^=59% (95%, CI 0 to 87) remained high (Figure 10).

Due to insufficient data and a large heterogeneity of outcomes, we could not perform similar analyses about other types of deregulated miRNAs and to determine the association between miR-146b and miR-221/221 clusters with other outcomes.

#### 3.6.3. Sensitivity Analysis by the Histological Subtypes of TC

From the total number of the studies, four studies [64,69,73,76] involving only PTC patients evaluated the association of all upregulated miRNAs with distant and regional recurrence. The analyzed miRNAs (miR-146a, -146b, -155, -193b, -221, -222, and -1299) showed a significant prognostic impact with an OR of 8.40, 95% CI 1.79 to 39.37 (log OR = 2.21, 95% CI 0.58 to 3.67, *p* < 0.001), and high heterogeneity, I^2^ = 80% (95% CI: 0 to 90). 

Regarding DFS/RFS, studies evaluating the PTC subtype [58,61,74,77] relieved a non-significant association between reported upregulated miRNAs (miR-146b, -203, -220, -221, and -222), (*p* > 0.005) and a significant association with the downregulated miRNAs (miR-9, -10b, -21, and -26a), with a pooled HR of 0.65 95%, CI 0.50 to 0.84 (log HR = −0.42, 95% CI: −0.67 to −0.17), and high heterogeneity, I^2^ = 68% (95% CI: 0 to 89). 

Regarding OS, when we have restricted the analysis to studies with PTC patients, the negative impact of the upregulated (miR-182, miR-203) and downregulated miRNAs (miR-26a, -381, and -791) on survival continued to be significant, with an HR of 4.23, 95%, CI 1.84 to 9.70 (log HR of 1.44, 95% CI 0.61 to 2.27), and low heterogeneity I^2^ = 8% (95% CI: 0 to 60), and respectively an HR of 0.38 95%, CI: 0.28 to 0.52 (log HR = −0.95, 95% CI: −1.27 to −0.63), and high heterogeneity, I^2^ = 70% (95% CI: 0 to 90) [59,62,72,74,75]. 

Besides, we have conducted sensitivity analyses for specific miRNAs (miR-146b, miR-221, and miR-222) and subtypes of TC regarding recurrence as the outcome of interest. Thus, estimations for mir-146b, identified only in studies involving patients with PTC, revealed a significant association with recurrence (OR = 9.11, 95% CI: 3.00 to 27.52; log OR = 2.21, 95% CI: 1.12 to 3.30, *p* < 0.001) and a reduced amount of heterogeneity I^2^ = 59% (95%, CI: 0 to 87) (Figure 9). Looking only at the mir-221, we have found a significant association with an OR of 3.88 (95% CI: 1.34 to 11.19, log OR = 1.35, 95% CI: 0.29 to 2.41, *p* < 0.001), and an average amount of heterogeneity I^2^ = 34% (95% CI: 0 to 67). Similar results regarding PTC were revealed when we estimated for miR-222 an OR of 6.56 (95% CI: 2.75 to 15.64, log OR = 1.88, 95% CI: 1.01 to 2.75, *p* < 0.001), I^2^ = 0% as well as for miR-221/222 as a family, an OR of 4.93 (95% CI 2.09 to 11.66; log OR = 1.59, 95% CI 0.73 to 2.45, *p* < 0.001), I^2^ = 0%. 

**Figure 11:**
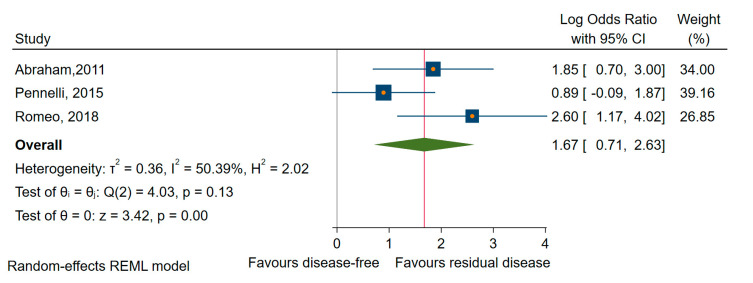
Forest plot of the association between the upregulated miRNAs and residual disease in MTC.

To identify the impact of dysregulated miRNAs in MTC, we performed a meta-analysis on the outcome of residual and persistent disease (a subcategory of the first) [42,68,78]. We found that higher levels of miRNAs (miR-21, -183, and -375) were significantly associated with residual disease (OR = 3.74, 95% CI 1.47 to 9.48; log OR = 1.32, 95% CI: 0.39 to 2.25, *p* < 0.005) and moderate heterogeneity, I^2^ = 34% (95% CI: 0 to 67) (Figure 11).

Due to insufficient data, we could not perform similar analyses about other histological subtypes, such as FTC, PDTC, and ATC.

## 4. Discussion 

In this systematic review and meta-analysis, we aimed to explore the utility of miRNA biomarkers that can be efficiently and robustly evaluated in predicting prognosis in TC. We searched the four central publication databases to ensure the identification of all relevant publications. To our knowledge, this is the first extensive meta-analysis, including a wide range of miRNAs from both tissue and serum samples, to describe the prognostic role of miRNAs.

The evidence in this review mostly applies to newly diagnosed patients with TC who received initial curative therapy. The studies included in this review addressed our research question in a total of 2011 participants, evaluating 39 miRNAs. Overall, the findings from this review support the statement that in this group of individuals, specific dysregulated miRNAs can predict long-term prognostic outcomes in TC. 

Initially, all the miRNAs were combined in a few meta-analyses, based on the type of dysregulation, to observe their impact on the primary endpoints of poor prognosis. The findings emerging from these meta-analyses suggest that the up- and downregulated miRNAs were significantly correlated with a decreased OS and DFS/RFS. Moreover, the upregulated miRNAs were associated with a higher recurrence rate. 

After this, a few select miRNAs, miR-146b, miR-221, and miR-222 were individually assessed to identify and highlight the specific miRNA, which may have potential prognostic value. The sensitivity analysis for recurrence shows a considerable disadvantage for participants with an overexpression of miR-146b and miR-221/222 family members in follicular cell-derived DTC (PTC and FTC). The findings emerging from the stratified analysis by miR-221/222 family member types revealed that elevated expression levels of miR-221 and miR-222 were significantly associated with recurrence and suggested their possible prognostic ability. Although various other miRNAs have been associated with prognosis in TC, most of these miRNAs were assessed in only a single study.

Third, we computed a sensitivity analysis concerning specific histological subtypes of TC to evaluate the specific prognostic role of the miRNAs. Accordingly, when considering the regional and distant recurrence as the outcome of interest, the upregulated miRNAs, specifically miR-146b, miR-221, and miR-222, were significantly associated with an increased risk of recurrence in PTC subjects. Moreover, two upregulated (miR-182 and miR-203) and three downregulated miRNAs (miR-26a, -381, and -791) could predict OS in PTC patients. However, the downregulated miRNAs (miR-9, -10b, -21, and -26a) in contrast to several upregulated miRNAs (miR-146b, -203, -221, and -222) could predict significantly the decreased DFS/RFS in PTC patients. Concerning MTC, we found that the pooled OR for residual disease indicates a prognostic value of specific upregulated miRNAs (miR-21, miR-183, and miR-375) in this subtype of TC. 

Unfortunately, due to insufficient data, we could not perform similar analyses involving other histological subtypes, such as FTC, PDTC, ATC, and other types of miRNAs.

These results are consistent with previous observations based on experimental studies, which show that miR-146b promotes cell migration and invasion with the expression of cancer-promoting genes and regulators of apoptosis [58]. Several clinical studies have suggested that elevated miR-146b expression may play a role in advanced malignant tumor characteristics, including extra-thyroidal invasion and advanced stages of PTC [91,92,93]. Furthermore, several recent research studies, including our own, have provided evidence that miR-146b overexpression even plays a critical role in PTC patients’ prognosis. MiR-146b levels were higher in Hürthle and tall cell papillary carcinoma, suggesting an association of these miRNAs with histologies of worse prognosis [79]. Thus, miR-146b has the chance to become a new marker in PTC outcome, associated with a malignant phenotype, as its deregulation occurs almost exclusively in TC [94]. 

MiR-221 and miR-222 are other highly overexpressed miRNAs in different follicular cell-derived TCs, similar to findings regarding miR-146b [94]. In vitro studies have identified that the miR-221/222 cluster regulates cell cycle and apoptosis downstream of the mitogen-activated protein kinase (MAPK) pathway; thus, its deregulation has been associated with treatment resistance, recurrence, worse prognosis, and aggressive disease behavior [30]. According to tumor histology, miR-222 levels were found to be twofold to threefold higher in tall cell papillary carcinoma than in the rest of histologies, suggesting an association with a worse prognosis [79]. Given the results of our meta-analysis, the miR-221/222 cluster has the chance to become a novel biomarker of recurrence in PTC patients.

Similar results concerning MTC have been reported in the previous studies, which confirmed the presence of significant miR-183 overexpression in lymph nodes of patients with MTC, knowing that lymph node involvement is one of the most important prognostic factors for poor survival in MTC [95]. At the same time, experiments on thyroid follicular cell line cultures demonstrated that miR-183 overexpression stimulates migration and led to a reduced apoptosis rate [96]. Some research has shown an opposing dual oncogenic potential of miR-183 explained by the target tissue type and mRNA targets expressed in that specific tissue. Therefore, the miR-183 overexpression has been implicated in the pathogenesis of various neoplasms such as hepatocellular carcinoma [97], melanoma [98], and colorectal cancer [99]. However, it also appears to have a suppressive effect on long-term cancer cells [100]. 

We showed that upregulated miR-375 is associated with residual disease in MTC patients, advocating its involvement in the prognosis of the disease. Several previous studies have already proven that miR-375 is indeed upregulated in MTC, indicating that it might play a central role in the tumorigenesis of MTC, via targeting multiple crucial pathways, mainly the phosphatidylinositol 3-kinase/ serine/threonine protein kinase B (PI3K/Akt) pathway [101]. So far, SEC23A is the only validated target gene of miR-375 in MTC [102].

We have to consider some limitations when interpreting the results of the current study. First, the number of available studies was limited, especially those evaluating serum miRNAs as circulating biomarkers, as they can be assayed before surgery and monitored throughout the lifespan, and they could be more valuable than tissue biomarkers. Second, small numbers of patients were analyzed in each study, raising questions about achieving adequate statistical power. Third, we observed a marked heterogeneity in some of the analyses, which is partly due to differences in patient characteristics, the use of different assay methods, endogenous normalization control, cut-off values for miRNA expression levels, follow-up durations, multiple outcomes, and effect sizes. Fourth, a low to moderate overall methodological quality of the included studies could have led to imprecise assumptions. Fifth, the absence of studies to cover the more aggressive histological subtypes, such as ATC or PDTC, make our findings of the potential prognostic role of the miR-146b, miR-221/222 cluster be assigned to just PTC and minimally invasive FTC. 

If comparing the miRNAs versus traditional surveillance biomarkers, Thyroglobulin (Tg), Calcitonin (Ctn), and Carcinoembryonic Antigen (CEA), there are concerns about inter-laboratory assay variability, the cost–benefit, and the accessibility constraints of the new biomarkers. Future feasibility studies and standardized protocol-based studies should approach these issues in the future. Despite these uncertainties, the serum miRNAs could replace or supplement Tg in long-term surveillance of the PTC patients who have had less than total thyroidectomy, in up to a quarter of PTC patients with high anti-Tg antibodies titers [103] or to those who have not had postoperative RAI [25]. 

Rosignolo et al. found that postoperative changes in circulating levels of miR-146a-5p and miR-221-3p in PTC patients display a good correlation with American Thyroid Association (ATA)-defined response-to-therapy classes, even in cases in which serum Tg assay results are unreliable or difficult to interpret. These findings suggest that serum levels of miR-146a-5p and miR-221-3p might be used as complementary biomarkers for the early noninvasive detection of persistent/recurrent PTC, particularly in the expanding population of patients undergoing more conservative management of PTC [87] Similarly, specific miRNAs can replace the Ctn in cases of non-secreting poorly differentiated MTC, in which the diagnosis and surveillance are often challenging and delayed [104]. 

Many DTC dedifferentiate and become radioactive iodine (RAI)-refractory with worse outcomes [105]. MiRNAs deregulation may play a role in TC dedifferentiation and resistance to RAI therapy. A in vitro study found that miR-146b binds to PAX8 and sodium/iodide symporter (NIS), leading to impaired protein translation and a subsequent reduction in iodide uptake [106]. Accordingly, the existence of the miR-146b/PAX8/NIS regulatory chain may be exploited therapeutically in the future to modulate thyroid cell differentiation and iodide uptake for improved targeted treatment. Besides, with the knowledge that the activated MAPK signaling pathway suppresses the uptake of RAI, later studies investigating the use of miRNA-directed therapy that inhibit this pathway in the restoration of RAI sensitivity could be implemented [107].

Further efforts should focus on the collaborative research, with well-designed studies, rigorously reported, adhering to the Reporting Recommendations for Tumour Marker Prognostic Studies guidelines (REMARK) [108]. Moreover, the establishment of the miRNAs status as biomarkers and surrogate endpoints will be decided after the evaluation of their clinical *relevance* and *validity. Between now and then, the* comparison of miRNAs with currently available biomarkers, refinement of the assay technology, evaluation of the sensitivity, specificity, and cut-off values, testing their performance in PDTC and ATC will be the next steps to accomplish. Furthermore, there is a need for a complete understanding of the miRNAs implication in the molecular signaling pathways and their downstream targets. 

As an increasingly sophisticated biological model of TC is developed, it is clear that the measurement of miRNA expression might facilitate and optimize the management of patients with TC. Moreover, the integration of these molecular markers together with clinicopathological factors into a complex prognostication system may enable better predictions, assuming that every single biomarker plays a small role in the summative outcome of interest. The development of a safe and specific method to implement miRNA-targeted therapy would help improve the treatment of unresponsive TC. Given the limited research available, the clinical application of these findings has yet to be verified. Large-scale standardized protocol-based studies are required to improve the accuracy and reduce the bias.

## 5. Conclusions

This research supports the statement that specific up- and downregulated miRNAs are associated with poor outcome on long-term surveillance. A specific miRNA signature composed of miR-146b, miR-221, and miR-222 could serve as potential prognostic biomarkers of recurrence in TC patients, particularly in PTC. The evidence on the ability of the aforementioned microRNAs expression to distinguish between individuals with poor versus good prognosis may enable better predictions and aid decision-making for clinicians; they might facilitate and optimize the surgical management of patients with TC and could be integrated into a complex prognostication system in the future. However, given the limited research available, further studies are needed to strengthen these findings and sustain their clinical applicability.

## 6. Differences Between Protocol and Review

We could not perform a large part of the planned subgroup and sensitivity analyses, based on study design (e.g., prospective versus retrospective), population demographics (e.g., age, gender, nationality), disease stage (e.g., early versus advanced stage), types of biological samples (e.g., tissue versus serum/plasma), mutational status (e.g., BRAF versus TERT versus RET/PTC), and initial curative therapy (e.g., thyroidectomy with adjuvant RAI ablation versus thyroidectomy without adjuvant RAI), due to a limited reporting of studies and a small number of studies.

## Figures and Tables

**Table 1:** Characteristics of the included studies.

First Author, Year, Reference	Country	TC Subtype	Sample	Follow-Up, Months	Age	Female (%)	Number	Assay	Control	Cut-Off	miRNA, Expression	Outcome
Abraham, 2011 [42]	Australia	MTC	thyroid	82.8	51.8	46	44	qRT-PCR	RNU48	None	185↑, 375↑	D/R-Recurrence
												Residual disease
Buda, 2012 [54]	Israel	PTC	thyroid	60	51.8	100	8	qRT-PCR	RNU6	None	155↑, 15a↑, 19b↑, 200a↑, 21↑, 483-5p↓	Recurrence
Cavedon, 2017 [53]	Italy	MTC	thyroid	40	58.5	59	121	qRT-PCR	RNU6B	None	224↓	Persistence
							133					Progression
Chen, 2019 [57]	China	PTC	thyroid	60	N/A	63	44	qRT-PCR	RNU6	N/A	1271↓	OS
Chou, 2013 [58]	Taiwan	PTC	thyroid	127	43.7	70	71	qRT-PCR	RNU6	Median	146b↑	DFS
Dai, 2017 [61]	China	PTC	thyroid	68	45.8	76	78	qRT-PCR	RNU48	Median	146b↑, 21↓, 220↑, 221↑, 222↑, 9↓	RFS
Dettmer, 2014 [56]	Switzerland	PD+oPD	thyroid	<202	65.4/71.5	62	27	Microarray	RNU44RNU6	0.2-fold	150↑	TSS
				<82.7						0.5-fold	23b↑	DFS
Galuppini, 2017 [60]	Italy	MTC	thyroid	39	58	60	130	qRT-PCR	RNU6B	None	375↑	Progression
Gao, 2018 [59]	China	PTC	thyroid	80	N/A	N/A	160	qRT-PCR	RNU6	N/A	791↓	OS
Huang, 2017 [62]	China	PTC	serum	60	N/A	79	87	qRT-PCR	N/A	Median	381↓	OS
Jikuzono, 2013 [63]	Japan	MI-FTC	thyroid	120	47.2	67	34	qRT-PCR	RNU44	None	10b↑, 221↑, 221*↑, 222↑, 222*↑, 375↑, 92a↑	D-Recurrence
Lee, 2013 [64]	Australia	PTC	thyroid	40.6	57/44	69	26	qRT-PCR	RNU48	None	1299↑, 146b↑, 155↑, 193b↑, 221↑, 222↑	Recurrence
Liu, 2017 [65]	China	TC	thyroid	60	45.3	67	131	qRT-PCR	GAPDH	ROC (0.87-fold)	let 7a↓	OS
Liu, Ch., 2017 [66]	China	PTC	thyroid	N/A	N/A	78	136	qRT-PCR	RNU6	None	199a-3p↓	R-Recurrence
Mian, 2012 [67]	Italy	MTC	thyroid	48	60	40	40	qRT-PCR	RNU6	None	224↓	Persistence
Montero, 2019 [55]	Spain	DTC	thyroid	96	51.1	N/A	24	MiRNome profiling	N/A	median	139-5p↓	DFS
				36			60			None		Residual disease
Pennelli, 2015 [68]	Italy	MTC	thyroid	48	59.1	56	57	qRT-PCR	RNU6B	None	21↑	Persistence
Qiu, 2017 [69]	China	PTC	thyroid	12	38-67	53	73	qRT-PCR	Beta-actin	N/A	146a↑146b↑	OS
										None		Recurrence
Ren, 2017 [75]	China	PTC	serum	60	N/A	61	84	qRT-PCR	RNU6	Mean	26a↓	DFS
												OS
Romeo, 2018 [78]	Italy	mMTC	plasma	36	50/48	41	31	qRT-PCR	RNU6B	Median	375↑	OS
				65			45			None		Residual disease
Sondermann, 2015 [77]	Brazil	PTC	thyroid	120	46.9/46.5	83	66	qRT-PCR	RNU48	median	10b↓, 146b↑, 21↓, 9↓	LNM-RFS
Sun, 2019 [70]	China	PTC	thyroid	< 60	N/A	51	56	qRT-PCR	RNU6	Mean	486↓	OS
Wu, 2019 [71]	China	PTC	thyroid	< 60	N/A	52	51	qRT-PCR	RNU6	Mean	26a↓	OS
Yao, 2019 [72]	China	PTC	thyroid	60	N/A	55	151	qRT-PCR	RNU6	Median	182↑	OS
Yip, 2011 [76]	USA	PTC	thyroid	73.2	42/44	76	32	qRT-PCR	RNU44	None	1↓, 130-b↓, 138↓, 146b↑, 155↑, 221↑, 222↑, 31↓, 34b↓	Recurrence
Zhang, 2017 [73]	China	PTC	serum	52	49.7/47.7	61	21	qRT-PCR	miR-16	None	146b↑, 221↑, 222↑	Recurrence
Zheng, 2017 [74]	China	PTC	serum	60	45.8/48.7	68	165	qRT-PCR	GAPDH	ROC (3.56-fold)	203↑	OS, RFS

Abbreviations: ↑= upregulated;↑=downregulated; DFS=disease-free survival; DTC=differentiated thyroid cancer; HR=hazard ratio; LNM=lymph node metastasis; mMTC=metastatic MTC; D-recurrence = distant recurrence; MTC=medullary thyroid cancer; N/A=not available; OR=odds ratio; OS=overall survival; PDTC=poorly differentiated thyroid cancer; PTC=papillary thyroid cancer; qRT-PCR=Quantitative Reverse Transcription–Polymerase Chain Reaction; RFS=recurrence-free survival; R- Recurrence=regional recurrence; ROC=receiver operating characteristic curve; SD=standard deviation; TC=thyroid cancer; TSS=tumor-specific survival.

**Table 2:** Characteristics of the upregulated miRNAs.

Upregulated miRNAs					
miRNA	Outcome	Analysis	HR/OR and 95% CI	Source	Study
10b	D-Recurrence	OR	19.8 (4.6–85.2)	Estimated	Jikuzono, 2013 [63]
15a	Recurrence	N/A	N/A	N/A	Buda, 2012 [54]
19b	Recurrence	N/A	N/A	N/A	Buda, 2012 [54]
23b	DFS	HR	2.6 (1.0–6.7)	Provided	Dettmer, 2014 [56]
92a	D-Recurrence	OR	7.4(1.9–29.2)	Estimated	Jikuzono, 2013 [63]
146a	OS.	N/A	N/A	N/A	Qiu, 2017 [69]
	Recurrence	OR	92.5 (27.0–315.8)	Estimated	Qiu, 2017 [69]
146b	DFS	HR	3.9 (1.7–8.8)	Provided	Chou, 2013 [58]
	LNM-RFS	HR	0.9 (0.7–1.1)	Provided	Sondermann, 2015 [77]
	OS.	N/A	N/A	N/A	Qiu, 2017 [69]
	Recurrence	OR	4.0 (0.8–18.1)	Estimated	Lee, 2013 [64]
	Recurrence	OR	36.5 (11.6–114.8)	Estimated	Qiu, 2017 [69]
	Recurrence	OR	7.9 (2.0–30.7)	Estimated	Yip, 2011 [76]
	Recurrence	OR	4.1 (0.8–20.9)	Estimated	Zhang, 2017 [73]
	RFS	HR	1.1 (0.2–4.6)	Provided	Dai, 2017 [61]
150	TSS	HR	5.0 (1.2–19.6)	Provided	Dettmer, 2014 [56]
155	Recurrence	N/A	N/A	N/A	Buda, 2012 [54]
	Recurrence	OR	1.5 (0.3–6.7)	Estimated	Lee, 2013 [64]
	Recurrence	OR	1.5 (0.4–5.3)	Estimated	Yip, 2011 [54]
182	OS	HR	2.8 (0.9–8.3)	Provided	Yao, 2019 [72]
183	D-Recurrence	OR	7.3 (1.9-26.9)	Estimated	Abraham, 2011 [42]
	R-Recurrence	OR	7.5 (2.2–24.7)	Estimated	Abraham, 2011 [42]
	residual disease	OR	7.0 (2.2–22.4)	Estimated	Abraham, 2011 [42]
193b	Recurrence	OR	1.2 (0.2–5.4)	Estimated	Lee, 2013 [64]
200a	Recurrence	N/A	N/A	N/A	Buda, 2012 [54]
203	OS	HR	6.7 (2.0–22.1)	Provided	Zheng, 2017 [74]
	RFS	HR	1.38 (1.0–1.7)	Provided	Zheng, 2017 [74]
220	RFS	HR	1.1 (0.3–3.4)	Provided	Dai, 2017 [61]
221	D-Recurrence	OR	7.9 2.0–31.0	Estimated	Jikuzono, 2013 [63]
	RFS	HR	1.4 (1.1–1.8)	Provided	Dai, 2017 [61]
	Recurrence	OR	2.2 (0.5–9.7)	Estimated	Lee, 2013 [64]
	Recurrence	OR	2.6 (0.7–9.4)	Estimated	Yip, 2011 [76]
	Recurrence	OR	14.4 (2.4–84.2)	Estimated	Zhang, 2017 [73]
221*	D-Recurrence	OR	8.0 (2.0–31.8)	Estimated	Jikuzono, 2013 [63]
222	D-Recurrence	OR	8.9 (2.2-35.4)	Estimated	Jikuzono, 2013 [63]
	Recurrence	OR	5.7 (1.2–26.8)	Estimated	Lee, 2013 [64]
	Recurrence	OR	5.0 (1.3–18.7)	Estimated	Yip, 2011 [76]
	Recurrence	OR	12.4 (2.1–70.8)	Estimated	Zhang, 2017 [73]
	RFS	HR	2.8 (1.1–7.1)	Provided	Dai, 2017 [61]
222*	D-Recurrence	OR	13.0 (3.1–53.8)	Estimated	Jikuzono, 2013 [63]
375	D-Recurrence	OR	9.3 (2.4–35.0)	Estimated	Abraham, 2011 [42]
	R-Recurrence	OR	7.5 (2.2–24.7)	Estimated	Abraham, 2011 [42]
	residual disease	OR	5.6 (1.8–17.8)	Estimated	Abraham, 2011 [42]
	Progression	OR	3.4 (1.2–9.9)	Estimated	Galuppini, 2017 [60]
	D-Recurrence	OR	2.4 (0.6–9.0)	Estimated	Jikuzono, 2013 [63]
	OS	HR	10.6 (3.8–29.5)	Provided	Romeo, 2018 [78]
	residual disease	OR	13.4 (3.2–55.9)	Estimated	Romeo, 2018 [78]
1299	Recurrence	OR	1.7 (0.4–7.6)	Estimated	Lee, 2013 [64]

Abbreviation: DFS = disease-free survival; HR = hazard ratio; LNM = lymph node metastasis; D-Recurrence = distant recurrence; N/A = not available; OR = odds ratio; OS = overall survival; RFS = recurrence-free survival; Recurrence = metastatic recurrence; TSS = tumor-specific survival

**Table 3:** Characteristics of downregulated miRNAs.

Downregulated miRNAs					
miRNA	Outcome	Analysis	HR/OR and 95% CI	Source	Study
1	Recurrence	OR	2.5 (0.7–9.1)	Estimated	Yip, 2011 [76]
9	RFS	HR	1.3 (0.4–3.8)	Provided	Dai, 2017 [61]
	LNM-RFS	HR	1.4 (1.2–1.7)	Estimated	Sondermann, 2015 [77]
10b	LNM-RFS	HR	1.2 (0.8–1.8)	Provided	Sondermann, 2015 [77]
26a	DFS	HR	2.8 (1.5–5.1)	Provided	Ren, 2017 [75]
	OS	HR	2.5 (1.3–4.8)	Provided	Ren, 2017 [75]
	OS.	N/A	N/A	N/A	Wu, 2019 [71]
31	Recurrence	OR	1.8 (0.5–6.7)	Estimated	Yip, 2019 [76]
34b	Recurrence	OR	5.0 (1.3–18.9)	Estimated	Zhang, 2017 [73]
130-b	Recurrence	OR	4.8 (1.3–18.1)	Estimated	Yip, 2011 [76]
138	Recurrence	OR	2.3 (0.6–8.5)	Estimated	Yip, 2011 [76]
139-5p	DFS	HR	0.2 (0.1–0.4)	Estimated	Montero, 2019 [55]
	Residual disease	OR	7.0 (2.6–18.9)	Estimated	Montero, 2019 [55]
199a-3p	R-Recurrence	OR	3.3 (1.1–9.8)	Estimated	Liu, Ch., 2017 [66]
224	Persistence	OR	3.4 (1.6–7.0)	Estimated	Cavedon, 2017 [53]
	Persistence	OR	4.7 (1.4–15.3)	Estimated	Mian, 2012 [67]
	Progression	OR	0.7 (0.5–0.9)	Provided	Cavedon, 2017 [53]
381	OS	HR	4.7 (2.6–8.5)	Provided	Huang, 2017 [62]
483-5p	Recurrence	N/A	N/A	N/A	Buda, 2012 [54]
486	OS	N/A	N/A	N/A	Sun, 2019 [70]
791	OS	HR	0.5 (0.3–0.9)	Provided	Gao, 2018 [59]
1271	OS	N/A	N/A	N/A	Chen, 2019 [57]
let 7a	OS	HR	0.4 (0.2–0.9)	Provided	Liu, 2017 [65]

Abbreviations: DFS = disease-free survival; HR = hazard ratio; LNM = lymph node metastasis; N/A = not available; OR = odds ratio; OS = overall survival; RFS = recurrence-free survival.

**Table 4:** Characteristics of the miRNAs with inconsistent direction.

MiRNAs with Inconsistent Expression Direction						
miRNA	↑/↓	Outcome	Analysis	HR/OR and 95% CI	Source	Study
21	↑	Recurrence	N/A	N/A	N/A	Buda, 2012 [54]
	↑	Persistence	OR	2.4 (0.9–6.5)	Estimated	Pennelli, 2015 [68]
	↓	RFS	HR	2.0 (0.4–8.1)	Provided	Dai, 2017 [61]
	↓	LNM-RFS	HR	1.5 (1.1–1.9)	Provided	Sondermann, 2015 [77]

Abbreviations: ↑ = upregulated; ↑ = downregulated; HR = hazard ratio; LNM = lymph node metastasis; OR = odds ratio; RFS = recurrence-free survival.

## Appendix A

**Table A1:** Quality Assessment of Prognostic Accuracy Studies (QUAPAS) questionaire.

Domain	Description	Signaling Question (Yes, No, Unclear)	Risk of Bias (High, Low, Unclear)	Concerns about Applicability (High, Low, Unclear)
**Participant recruitment**	Describe the method for recruiting participants. Describe participants (previous testing, presentation, the intended use of index test and setting)	Was there consecutive or random enrollment of participants? Do the participants represent the intended population? Did the study avoid inappropriate exclusions?	Could the selection of participants have introduced bias?	Are there concerns that the participants do not match the review question?
**Index test**	Describe index test (definition, method of measurement, interpretation)	Was the method and settings for performing the index test valid and reliable? Was the method and settings for performing the index test the same for all participants? If a threshold was used, was it prespecified?	Could the conduct or interpretation of the index test have introduced bias?	Are there concerns that the index test, its conduct, or its interpretation differ from the review question?
**Target event**	A clear definition of outcome is provided, including the duration of follow-up and level and extent of the outcome construct.	Was a clear definition of the outcome provided? Was the method used to measure the target event valid and reliable? Was the method used to measure the target event the same for all participants? Was the target event measured without knowledge of the index test results?	Could the measurement of the target event have introduced bias?	Are there concerns that the target event does not match the review question?
**Study flow**	Describe the time horizon from the index test to the target event. Describe any participants lost to follow-up or excluded from the 2x2 table.	Was the information on the target event available for all participants? Is the loss to follow-up related to the test results?	Could the study flow have introduced bias?	Are there concerns that the time horizon does not match the review question?
**Analysis**	Describe the statistical methods	Were the methods used to account for censoring? Was the statistical method appropriate for the design of the study? Were methods used to account for competing events?	Could analysis have introduced bias?	

## Appendix B

**Table A2:** ‘Risk of bias’ and ‘Applicability concerns’ assessment according to QUAPAS by the outcome.

Type of Studies	Study, Reference	Risk of Bias					Applicability Concerns			
		Patient Selection	Index Test	TArget Event	Study Flow	Analysis	Patient Selection	Index Test	Target Event	Study Flow
**Studies reporting time-to-event outcomes**	Chen [57]	↑	?	?	↑	↑	↓	↓	?	↓
	Chou [58]	↑	↓	↓	↑	↓	↓	↓	↓	↓
	Dai [61]	↓	↓	↓	↓	↓	↓	↓	↓	↓
	Dettmer [56]	↑	↓	?	↓	↓	↑	↑	↓	↑
	Gao [59]	↑	?	?	↑	↓	?	↓	?	↓
	Huang [62]	↓	↓	?	?	↓	↓	↓	?	?
	Liu [65]	↓	↓	?	↓	↓	↑	↓	↓	↓
	Montero [55]	↑	↓	?	↑	↑	↓	↑	?	↓
	Qiu [69]	?	?	?	↑	↑	↓	↓	?	↑
	Ren [75]	?	↓	?	↓	↓	↓	↓	?	↓
	Romeo [78]	↑	↓	?	↑	↓	↓	↓	↓	↓
	Sondermann [77]	↑	↓	?	↓	↓	↓	↓	↓	↓
	Sun [70]	↑	↓	?	↑	↑	↑	↓	?	↓
	Wu [71]	↓	↓	?	↑	↑	↑	↓	?	↓
	Yao [72]	↑	↓	?	↑	↓	↑	↓	?	↓
	Zheng [74]	↑	?	?	↓	↓	↑	↓	?	↓
**Studies reporting other parameters of** **TC aggressive behavior**	Abraham [42]	?	↓	?	↑	↑	?	↓	↓	↓
	Buda [54]	↑	↓	↑	↑	↑	↑	↓	?	↓
	Cavedon [53]	↓	↓	?	?	↑	↓	↓	↓	↑
	Galuppini [60]	↓	↓	?	↓	↑	?	↓	↓	↑
	Jikuzono [63]	↑	↓	?	↓	↑	↑	↓	↑	↓
	Lee [64]	↓	↓	?	↓	↓	↑	↓	↓	↑
	Liu, Ch. [66]	?	↓	↑	↓	↑	↑	↓	↑	?
	Mian [67]	↓	↓	↑	↓	↑	?	↓	↑	↑
	Montero [55]	↑	↓	?	?	↑	↓	↑	↓	↑
	Pennelli [68]	↓	↓	?	↓	↑	?	↓	↓	↓
	Qiu [69]	?	↓	↑	↓	↓	↓	↓	↓	↑
	Romeo [78]	↑	↓	?	↓	↑	↓	↓	↓	↑
	Yip [76]	↑	↓	↑	↓	↓	↓	↓	?	?
	Zhang [73]	↑	↓	?	↓	↑	↑	↓	↓	↑
	↓ Low-Risk ↑ High Risk ? Unclear Risk									
